# The Association of Postprandial Triglyceride Variability with Renal Dysfunction and Microalbuminuria in Patients with Type 2 Diabetic Mellitus: A Retrospective and Observational Study

**DOI:** 10.1155/2022/3157841

**Published:** 2022-01-10

**Authors:** Natsumi Matsuoka-Uchiyama, Haruhito A. Uchida, Shugo Okamoto, Yasuhiro Onishi, Katsuyoshi Katayama, Mariko Tsuchida-Nishiwaki, Hidemi Takeuchi, Rika Takemoto, Yoshiko Hada, Ryoko Umebayashi, Naoko Kurooka, Kenji Tsuji, Jun Eguchi, Hirofumi Nakajima, Kenichi Shikata, Jun Wada

**Affiliations:** ^1^Department of Nephrology, Rheumatology, Endocrinology and Metabolism, Okayama University Academic Field of Medicine, Dentistry, and Pharmaceutical Sciences, Okayama, Japan; ^2^Department of Chronic Kidney Disease and Cardiovascular Disease, Okayama University Academic Field of Medicine, Dentistry and Pharmaceutical Sciences, Okayama, Japan; ^3^Center of Ultrasonic Diagnostics, Okayama University Hospital, Okayama, Japan; ^4^Nakashima Hospital, Okayama, Japan; ^5^Center for Innovative Clinical Medicine, Okayama University Hospital, Okayama, Japan

## Abstract

**Objective:**

We examined whether or not day-to-day variations in lipid profiles, especially triglyceride (TG) variability, were associated with the exacerbation of diabetic kidney disease.

**Methods:**

We conducted a retrospective and observational study. First, 527 patients with type 2 diabetes mellitus (DM) who had had their estimated glomerular filtration rate (eGFR) checked every 6 months since 2012 for over 5 years were registered. Variability in postprandial TG was determined using the standard deviation (SD), SD adjusted (Adj-SD) for the number of measurements, and maximum minus minimum difference (MMD) during the first three years of follow-up. The endpoint was a ≥40% decline from baseline in the eGFR, initiation of dialysis or death. Next, 181 patients who had no micro- or macroalbuminuria in February 2013 were selected from among the 527 patients for an analysis. The endpoint was the incidence of microalbuminuria, initiation of dialysis, or death.

**Results:**

Among the 527 participants, 110 reached a ≥40% decline from baseline in the eGFR or death. The renal survival was lower in the higher-SD, higher-Adj-SD, and higher-MMD groups than in the lower-SD, lower-Adj-SD, and lower-MMD groups, respectively (log-rank test *p* = 0.0073, 0.0059, and 0.0195, respectively). A lower SD, lower Adj-SD, and lower MMD were significantly associated with the renal survival in the adjusted model (hazard ratio, 1.62, 1.66, 1.59; 95% confidence intervals, 1.05-2.53, 1.08-2.58, 1.04-2.47, respectively). Next, among 181 participants, 108 developed microalbuminuria or death. The nonincidence of microalbuminuria was lower in the higher-SD, higher-Adj-SD, and higher-MMD groups than in the lower-SD, lower-Adj-SD, and lower-MMD groups, respectively (log-rank test *p* = 0.0241, 0.0352, and 0.0474, respectively).

**Conclusions:**

Postprandial TG variability is a novel risk factor for eGFR decline and the incidence of microalbuminuria in patients with type 2 DM.

## 1. Introduction

Diabetic mellitus (DM) is the most common cause of end-stage renal disease (ESRD) worldwide and is closely associated with increased cardiovascular risk and mortality (1) (2). The data from the United States Renal Data System showed that after a year-by-year rise in the number of incident ESRD cases from 1980 through 2000, the count plateaued between 2007 and 2011 but rose again from 2012 to 2017 due to the aging of the population and the increasing prevalence of obesity and DM (3). Thus, DM still presents a big problem that should be dealt with.

Recent epidemiological studies suggest that diabetic kidney disease (DKD) patients have a variety of clinical presentations and progression rates to ESRD although typical clinical manifestations of DKD are characterized by slow progression from microalbuminuria to macroalbuminuria and by hyperfiltration at the early stage and a progressive decline in the glomerular filtration rate (GFR) at the advanced stage. Some DKD patients lose their renal functions without albuminuria (4). This population is presumably related to atherosclerosis by aging, hypertension, and dyslipidemia. Furthermore, components of metabolic syndrome, like abdominal obesity, hypertension, hyperglycemia, and dyslipidemia, are highly interrelated and contribute to the development and progression of DKD (5).

In recent years, increasing evidence has suggested that not only the average blood pressure and blood glucose level but also the glycemic and blood pressure variability can be independent risk factors for development of albuminuria and a GFR in type 2 DM (6). Epidemiological data confirmed that a tight control of the glucose and blood pressure level is pivotal and modifiable key factors for preventing the incidence and progression of DKD (7) (8) (9).

Dyslipidemia, an element of metabolic syndrome, is linked to a reduction in the GFR and the development of albuminuria in patients with type 2 DM (10–13). Both fasting and postprandial hypertriglyceridemia have already been reported as being associated with a reduction in the estimated GFR (eGFR) and the development of albuminuria (14–16). Numerous clinical trials have revealed the importance of lipid control in preserving the GFR in patients with DM (17). However, whether or not lipid variability exacerbates DKD remains unclear, although several observational studies have suggested a possible impact of high-density lipoprotein cholesterol (HDL-C) variability and fasting triglyceride (TG) variability on the appearance of albuminuria (18, 19).

The present study investigated the association of intraindividual variability in postprandial TG with the eGFR decline and the incidence of microalbuminuria in type 2 DM to clarify whether or not postprandial visit-to-visit TG variability is associated with the exacerbation of DKD.

## 2. Materials and Methods

### 2.1. Study Design and Participants

**Figure 1:**
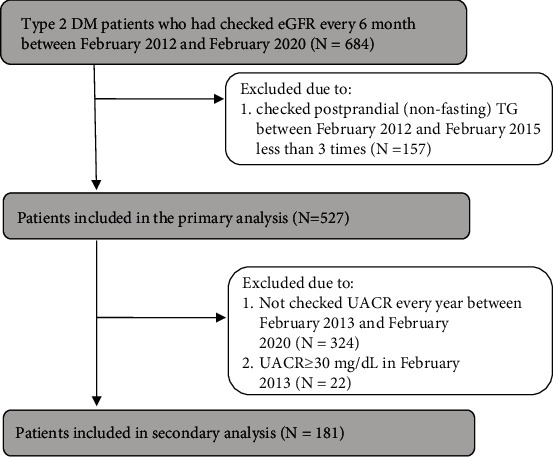
Study flow. After excluding subjects who did not meet our study criteria, a total of 527 participants were included in the primary analysis, and a total of 181 participants were included in the secondary analysis. DM: diabetic mellitus; eGFR: estimated glomerular filtration rate; TG: triglyceride; UACR: urine albumin-to-creatinine ratio.

A longitudinal, retrospective, observational cohort study was conducted to examine the association of visit-to-visit postprandial TG variability with the eGFR decline in patients, >20 years old, with type 2 DM who had had their eGFR checked every 6 months since February 2012 for over 5 years at a single hospital. Patients who regularly checked their renal function every February and August were registered. Those who checked their postprandial (nonfasting) TG fewer than three times between February 2012 and February 2015 were excluded. Data were collected up to the last observation in February 2020. The total number of participants was 527 patients, including 42 individuals who moved to other places for personal reasons with 5.5 to 7.5 years of follow-up. Those patients were included as censored data, and our analysis was based on the full analysis set (Figure 1).

Three indices of postprandial TG variability were calculated; the standard deviation (SD), SD adjusted (Adj-SD) for the number of measurements, and maximum minus minimum difference (MMD) of postprandial TG during the first three years of follow-up (19–22) (23, 24). To minimize the effect of different numbers of TG measurements on the calculated values, the Adj-SD was defined according to the following formula: Adj − SD = SD/√[*n*/(*n* − 1)]. The participants were separated into two groups by the median SD, Adj-SD, and MMD values. The primary endpoint was *a* ≥ 40% decline from the baseline eGFR, the initiation of dialysis or death.

We also extracted the participants who had had their urine albumin-to-creatinine ratio (UACR) checked every year between February 2013 and February 2020, over 7 years among the 527 total participants in order to analyze the association between postprandial TG variability and incidence of microalbuminuria in patients with type 2 DM. The patients who already had micro- or macroalbuminuria in 2013 were excluded, leaving a total of 181 such patients (Figure 1). These participants were also divided into two groups by the median SD, Adj-SD, and MMD values. The secondary endpoint was the incidence of microalbuminuria (UACR ≥ 30 mg/gCr), the initiation of dialysis, or death.

### 2.2. Anthropometric Measurements

The body mass index (BMI) was calculated as weight (kg) divided by height (m) squared.

### 2.3. Laboratory Measurements

The following patient characteristics were collected in 2012: age, sex, duration of DM, BMI, systolic and diastolic blood pressure, eGFR, serum creatinine, HDL-C, low-density lipoprotein cholesterol (LDL-C), HbA1c, smoking habit (current, past, never), proteinuria, and medication (statins, fibrates, and cholesterol transport inhibitors). The eGFR was calculated using the formula modified for Japanese subjects: eGFR (mL/min/1.73 m^2^) = 194 × serum creatinine (mg/dL)^−1.094^ × Age^−0.287^ (×0.739 for females) (25). Blood samples were collected two to six hours after breakfast or lunch as postprandial (nonfasting) samples. The mean TG is the average postprandial TG value between February 2012 and February 2015. Serum TG was assessed using an enzyme method (TG-EX, Denka®). The assay was performed within 24 h with an automated clinical chemistry analyzer. Urinary albumin excretion was tested using an immunonephelometric technique (TIA Micro Alb, Nittobo®).

### 2.4. Definition of Risk Factors and Covariates

DM was defined as glycated hemoglobin (HbA1c) ≥ 6.5% and fasting plasma glucose ≥ 126 mg/dL and/or postprandial plasma glucose ≥ 200 mg/dL, a self-reported history of DM or the use of any antidiabetes medication. Patients with type 1 DM or gestational diabetes were excluded. Regarding smoking status, current smokers were defined as participants who had a regular cigarette smoking habit in 2012, past smokers as those who had had a regular cigarette smoking habit and stopped smoking before 2012, and never smokers as those who had never smoked. Proteinuria was defined by urine dipstick tests using semiquantitative measurements ≥±. Statin, fibrate, and cholesterol transport inhibitor intake were defined based on the presence of a regular intake of such drugs in 2012.

The definition of risk factors for the multivariate Cox's proportional hazard regression model was as follows: (1) age, BMI, baseline eGFR, mean TG, and HbA1c as continuous variables; (2) proteinuria: urine dipstick tests by semiquantitative measure ≥±, 3) smoking habit: current smoker; (4) hypertension: systolic blood pressure ≥ 130 mmHg and/or diastolic blood pressure ≥ 80 mmHg; (5) fibrates intake: taking fibrates in 2012. All data were collected from the medical charts.

### 2.5. Statistical Analyses

Data were expressed as *n* (%) for categorical variables and the median (interquartile range) for continuous variables. A Kaplan–Meier analysis and Cox's proportional hazard regression model were adopted to calculate the cumulative probability to reach the endpoint and hazard ratio (HR) of eGFR decline and incidence of albuminuria. The estimated standard error of the confidence estimate was used to establish confidence intervals (CI) of the estimated HR. The statistical analyses were performed using the JMP software program, version 14.0.0 (SAS Institute, Inc., Cary, NC), and all *p* values were calculated as two-sided. The association was considered significant with *p* values less than 0.05.

## 3. Results

### 3.1. The Primary Analysis: eGFR Decline

#### 3.1.1. Characteristics

Baseline characteristics of the study participants for the primary analysis, divided by the median SD, Adj-SD, and MMD values, are listed in Table 1. The median SD, Adj-SD, and MMD values were 37, 34, and 95, respectively. The average number of times postprandial TG was measured between 2012 and 2015 was 5.5. Compared with the lower-SD, lower-Adj-SD, and lower-MMD groups, the patients in the higher-SD, higher-Adj-SD, and higher-MMD groups were significantly younger and had a shorter duration of DM, higher BMI, lower HDL-C, higher prevalence of proteinuria (≥±) and more patients who received fibrates or cholesterol transport inhibitors (Table 1). The TG levels were significantly higher in the group a higher variability in SD, Adj-SD, and MMD, than in the group with a lower variability.

#### 3.1.2. Clinical Outcomes

Among the 527 total participants, during a median follow-up of 8.0 years, 110 (21%) reached the primary endpoint. Ninety-two participants (17%) encountered an eGFR decline ≥ 40%, and 18 participants (3%) died. No participant needed dialysis. Sixty out of 203 participants with proteinuria (30%) reached the primary endpoint while 50 out of 324 without proteinuria (15%) reached the primary endpoint. The participants with proteinuria reached the primary endpoint significantly more often than those without proteinuria (HR, 2.30; 95% CI, 1.50 to 3.52).

**Figure 2:**
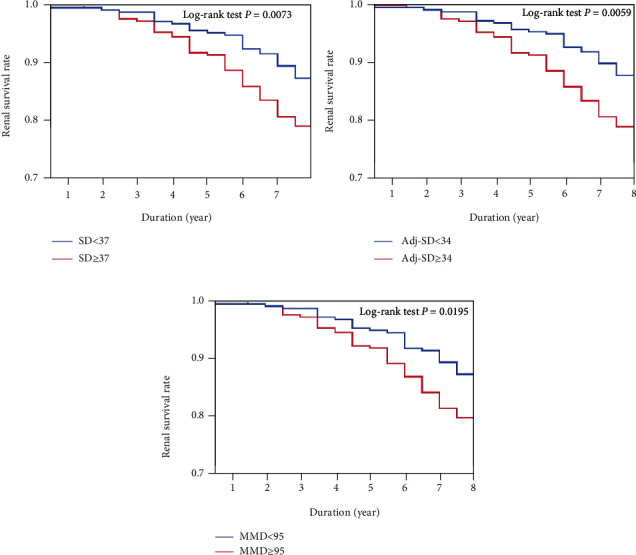
Comparing the eight-year renal survival rate between groups divided by the median values of SD, Adj-SD, and MMD. TG: triglyceride; SD: standard deviation; Adj-SD: SD adjusted for the number of measurements; MMD: maximum minus minimum difference.

The patients with an SD ≥ 37 demonstrated an 8-year renal survival rate of 73.7%, while the patients with an SD < 37 demonstrated a rate of 83.1%. The renal survival rate was thus lower in the group with an SD ≥ 37 than in the group with an SD < 37 (log-rank test *p* = 0.0073) (Figure 2). We performed a Cox's proportional hazard regression analysis of the baseline factors for a possible association with the renal survival. In this analysis, higher SD was significantly associated with the primary endpoint in the adjusted model (HR, 1.62; 95% CI, 1.05 to 2.53) (Table 2).

Next, regarding the Adj-SD, the 8-year renal survival rate was 73.5% in the group with an Adj − SD ≥ 34 and 83.1% in the group with an Adj − SD < 34. The renal survival rate was thus lower in the group with an Adj − SD ≥ 34 than in the group with an Adj − SD < 34 (log-rank test *p* = 0.0059) (Figure 2). According to a Cox's proportional hazard regression analysis, a higher Adj-SD was significantly associated with the primary endpoint in the adjusted model (HR, 1.66; 95% CI, 1.08 to 2.58) (Table 2).

Third, regarding the MMD, the 8-year renal survival rate was 74.3% in the group with an MMD ≥ 95 and 82.4% in the group with an MMD < 95. The renal survival rate was thus lower in the group with an MMD ≥ 95 than in the group with an MMD < 95 (log-rank test *p* = 0.0195) (Figure 2). According to a Cox's proportional hazard regression analysis, higher MMD was significantly associated with the primary endpoint in the adjusted model (HR, 1.59; 95% CI, 1.04 to 2.47) (Table 2).

### 3.2. The Secondary Analysis: Incidence of Microalbuminuria

#### 3.2.1. Characteristics

Baseline characteristics of the study participants for the secondary analysis, divided by the median SD, Adj-SD, and MMD values, are listed in Table 3. The median SD, Adj-SD, and MMD values were 37, 34, and 93, respectively. Compared with the lower-SD, lower-Adj-SD, and lower-MMD groups, the patients in the higher-SD, higher-Adj-SD, and higher-MMD groups had a significantly shorter duration of DM, lower HDL-C, and more patients who received fibrates and cholesterol transport inhibitors (Table 3).

#### 3.2.2. Clinical Outcomes

Among the 181 participants, 108 (60%) reached the secondary endpoint. One hundred and four participants (57%) encountered microalbuminuria, and 4 participants (2%) died. No participant needed dialysis.

**Figure 3:**
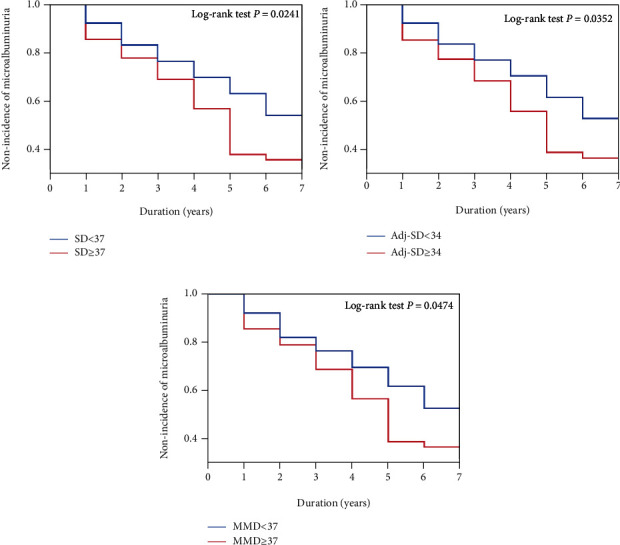
Comparing the seven-year nonincidence of microalbuminuria between groups divided by the median values of SD, Adj-SD, and MMD. TG: triglyceride; SD: standard deviation; Adj-SD: SD adjusted for the number of measurements; MMD: maximum minus minimum difference.

Regarding the SD, the 7-year nonincidence of microalbuminuria was 33.0% in the group with an SD ≥ 37 and 47.8% in the group with an SD < 37. The nonincidence of microalbuminuria was thus lower in the group with an SD ≥ 37 than in the group with an SD < 37 (log-rank test *p* = 0.0241) (Figure 3). We performed a Cox's proportional hazard regression analysis of the baseline factors with a possible association with the incidence of microalbuminuria. In this analysis, a higher SD was significantly associated with the secondary endpoint in the adjusted model (HR, 1.77; 95% CI, 1.08 to 2.88) (Table 4).

Next, regarding the Adj-SD, the 7-year nonincidence of microalbuminuria was 33.7% in the group with an Adj − SD ≥ 34 and 46.7% in the group with an Adj − SD < 34. The nonincidence of microalbuminuria was thus lower in the group with an Adj − SD ≥ 34 than in the group with an Adj − SD < 34 (log-rank test *p* = 0.0352) (Figure 3). According to a Cox's proportional hazard regression analysis, a higher Adj-SD was significantly associated with the secondary endpoint in the adjusted model (HR, 1.72; 95% CI, 1.05 to 2.81) (Table 4).

Third, regarding the MMD, the 7-year nonincidence of microalbuminuria was 34.1% in the group with an MMD ≥ 93 and 46.7% in the group with an MMD < 93. The nonincidence of microalbuminuria was thus lower in the group with an MMD ≥ 93 than in the group with an MMD < 93 (log-rank test *p* = 0.0474) (Figure 3). According to a Cox's proportional hazard regression analysis adjusted for age, sex, and the BMI (model 1), a higher MMD was significantly associated with the secondary endpoint in the adjusted model (HR, 1.49; 95% CI, 1.02 to 2.20); however, the significance of this association was diminished when further adjusted by the mean TG and/or baseline eGFR (model 2 and 3) (Table 4).

## 4. Discussion

In this study, the association of visit-to-visit TG variability with the eGFR decline and incidence of albuminuria in patients with type 2 DM was examined. The visit-to-visit variability of postprandial TG was a significant predictor of the eGFR decline in patients with type 2 DM during long-term follow-up, even when adjusting for confounding factors. In addition, the visit-to-visit variability of postprandial TG was also significantly associated with the incidence of microalbuminuria. Thus, we found that the visit-to-visit variability of postprandial TG was associated with DKD progression.

When considering “variability,” experimental models in vitro have shown that intermittent hyperglycemia is more detrimental for endothelial cells than continuous hyperglycemia (26). Glycemic variability is associated with the occurrence of various microvascular and macrovascular complications in DM because of excessive protein glycation end products and activation of oxidative stress in the causation of vascular complications (27, 28). In addition, blood pressure variability is also a significant prognostic factor in ESRD (29–31). Both the plasma glucose level and blood pressure naturally fluctuate to a certain extent and are components of metabolic syndrome, which are highly interrelated to the development and progression of DKD (5). Given these observations, a similar association may be found for variability of TG, which is another factor of metabolic syndrome and fluctuates to a certain extent. This is because it has already been reported that both fasting and postprandial hypertriglyceridemia are associated with the eGFR decline and incidence of albuminuria (14–16), the variability of fasting TG is predictive of coronary events (20), and the variability of fasting TG is also linked to the incidence of microalbuminuria in patients with type 2 DM (19).

While a device for measuring the trend in 24 h blood pressure and plasma glucose level has been developed, no such devise is available for the serum TG concentration. Therefore, we analyzed the postprandial TG variability by the SD, Adj-SD, and MMD. Previous papers on “variability” have used various indices to evaluate their variability. SD has been used to evaluate the glycemic, blood pressure, and fasting TG variability (19, 20, 32, 33) (34, 35); the Adj-SD has also been used to evaluate the glycemic and fasting TG variability with adjusting for the number of measurements (19, 21, 22, 36); and the MMD has been used to evaluate the glycemic and blood pressure variability (23, 24, 37, 38). Our study suggested that the SD and Adj-SD might be more reliable than MMD, but MMD is calculated more easily than the other two. In this sense, this method may be suitable for clinical situations. Using any of these approaches, the postprandial TG variability remains a significant risk factor for the eGFR decline and incidence of microalbuminuria in patients with type 2 DM.

It is important to determine the best timing for the measurement of TG in the clinical setting. The lipid profile is conventionally measured in plasma or serum obtained after fasting for at least eight hours and therefore may not reflect the daily average plasma lipid (39). In patients with DM, remnant lipoprotein cholesterol levels remain high throughout the day except for a few hours before breakfast (40). There is no evidence that fasting is superior to postprandial assessments when evaluating the lipid profile (41). The Danish Society for Clinical Biochemistry recommended that all laboratories in Denmark use random postprandial lipid profile measurements rather than fasting profiles (42). Traditionally, the Friedewald equation has been applied to a fasting lipid profile; however, the calculated level of LDL-C, which is determined using this equation at TG concentrations of ≤400 mg/dL, is similar to the LDL-C value measured directly on both fasting and postprandial lipid profiles (43, 44). In addition, numerous population-based studies and at least three major statin trials have used random, postprandial blood sampling, providing a robust evidence base for a change in the conventional practice of using fasting samples (45–47). Thus, there are both advantages and disadvantages in taking a postprandial lipid profile. In particular, in DM patients, both fasting and postprandial plasma glucose levels are important information to have for controlling DM in clinical practice, but fasting tests carry a hypoglycemic risk, and HbA1c can be accurately evaluated in either a fasting or nonfasting state.

The association between renal dysfunction and dyslipidemia had been described in the lipid nephrotoxicity hypothesis (48). There is evidence that the renal accumulation of lipids can cause structural and functional changes in mesangial cells, podocytes, and proximal tubule cells, which all contribute to the nephron function. Thus, it is widely recognized that ectopic deposition of lipids causes harm to target cells and organs; the ectopic accumulation of lipids in the kidney promotes maladaptive responses of renal cells to the mechanical forces of hyperfiltration, leading to podocyte depletion, proteinuria, focal segmental glomerulosclerosis, and interstitial fibrosis (49). Regarding lipid nephrotoxicity, our study adds a new significance regarding improved lipid management and thus achieves an optimal DKD treatment—namely, not only casual plasma TG concentrations but also TG variability should be controlled.

In our study, no significant difference in the baseline eGFR was found between the groups divided by the median values of SD, Adj-SD, and MMD. Although more patients with hyperfiltration might be included in the groups with higher SD, Adj-SD, and MMD values, the fact that the patients with a shorter renal survival demonstrated higher variability of postprandial TG remains important. It is also suggested that the patients with a higher peak TG value had a higher risk of DKD progression. Furthermore, the possibility that the pathophysiological mechanisms underlying postprandial TG variability also induce DKD progression, independent of TG levels, should be considered.

Several studies have demonstrated that postprandial hypertriglyceridemia is involved in the production of proinflammatory cytokines, recruitment of neutrophils, and generation of oxidative stress, resulting in endothelial dysfunction which is an initial process of atherogenesis and it might contribute to the development of albuminuria in healthy subjects as well as hypertriglyceridemic patients and type 2 DM patients (50–53). Our study showed that the patients with a higher variability of postprandial TG developed microalbuminuria significantly earlier than others, although no significant differences were found in the HbA1c, baseline UACR, or baseline eGFR values between the groups with higher and lower SD, Adj-SD, or MMD values. It is thus important to consider that microalbuminuria may develop earlier in type 2 DM patients with a higher variability of postprandial TG than in others.

Our study was not an interventional study, but an observational study. Therefore, further study is needed to clarify that lowering postprandial TG variability might be helpful for treating early-stage DKD to prevent its progression. A clinical study, the Fenofibrate Intervention and Event Lowering in Diabetes study, showed that fenofibrate prevented progression from normo-albuminuria to microalbuminuria in patients with type 2 DM (54–56). Fenofibrate in diabetic mice normalizes the endothelial function by balancing vascular reactivity via increasing nitric oxide production and suppressing the vasoconstrictor prostaglandin, suggesting a mechanism of action of fenofibrate (57). It has also been reported that ezetimibe, alogliptin, bezafibrate, vildagliptin, and omega-3 fatty acids improve postprandial hypertriglyceridemia and endothelial dysfunction; therefore, these medications have potential utility for preventing DKD progression (58–61) (62). Furthermore, a higher variability of postprandial TG may be a marker of incomplete or intermittent compliance with lifestyle measures. In patients with type 2 DM, the risk of kidney events tended to be decreased by multifactorial intensive treatment, including lipid control in addition to control of the glucose level and blood pressure (63). Given these observations, it may be wise to include care for visit-to-visit postprandial TG variability in lipid control efforts. A further follow-up interventional study is required to clarify the efficacy of lowering postprandial TG variability on delaying progression of DKD to ESRD.

### 4.1. Study Limitations

Several limitations of this study should be considered when interpreting its results. First, the sample size is relatively small, and this cohort was entirely enrolled from a single hospital by eight doctors. Second, this was a retrospective and observational study. Therefore, this study cannot state that lowering the visit-to-visit postprandial TG variability prevents progression of DKD. Third, most of the participants were at an early stage of DKD. Fourth, medication, treatment compliance, and diet might have affected the TG variability during the first three years of follow-up as this study was an observational study. Fifth, the effect of medication, treatment compliance, and diet during the observational period was not considered. Finally, the influence of alcohol intake on the day of or the day before the visit was not considered due to the fact that we did not have any data on whether the participants drink daily or not.

## 5. Conclusions

In conclusion, three different indices of postprandial visit-to-visit TG variability may be risk factors for eGFR decline and the incidence of microalbuminuria in patients with type 2 DM. The pathophysiological mechanisms underlying these associations and the effect of lowering the postprandial TG variability on preventing the progression of DKD remain to be further elucidated.

## Figures and Tables

**Table 1:** Characteristics of study participants for the primary analysis with three indices of postprandial TG variability groups (*N* = 527).

Variable	Higher-SD group: SD ≥ 37 (*n* = 266)	Lower-SD group: SD < 37 (*n* = 261)	*p* value	Higher-Adj-SD group: Adj − SD ≥ 34 (*n* = 260)	Lower-Adj-SD group: Adj − SD < 34 (*n* = 267)	*p* value	Higher-MMD group: MMD ≥ 95 (*n* = 264)	Lower-MMD group: SD < 95 (*n* = 263)	*p* value
Age (yr)	67 (60-75)	70 (63-77)	0.0014^∗^	67 (59-75)	70 (63-77)	0.0007^∗^	67 (59-75)	70 (63-77)	0.0013^∗^
Sex (male)	151 (57)	124 (48)	0.0334^∗∗^	147 (57)	128 (48)	0.0482^∗∗^	142 (54)	133 (51)	0.4597^∗∗^
Duration of diabetes (yr)	10 (5-17)	12 (7-19)	0.0157^∗^	10 (5-17)	12 (6-19)	0.0370^∗^	10 (5-16)	12 (7-19)	0.0126^∗^
BMI (kg/m^2^)	24.2 (22.2-26.4)	23.0 (21.0-25.1)	<0.0001^∗^	24.2 (22.2-26.5)	23.1 (21.0-25.1)	<0.0001^∗^	24.2 (22.1-26.5)	23.1 (21.1-25.1)	<0.0001^∗^
sBP (mmHg)	130 (122-140)	130 (122-138)	0.9196^∗^	130 (122-140)	130 (122-138)	0.7489^∗^	130 (122-138)	130 (122-138)	0.8013^∗^
dBP (mmHg)	78 (70-80)	76 (70-80)	0.0583^∗^	78 (70-80)	76 (70-80)	0.0356^∗^	78 (70-80)	76 (70-80)	0.0649^∗^
eGFR (mL/min/1.73 m^2^)	73.3 (60.4-85.7)	73.0 (60.5-85.2)	0.5317^∗^	73.5 (60.4-85.7)	72.6 (60.5-85.1)	0.4393^∗^	73.2 (60.1-87.4)	73.0 (60.6-84.4)	0.6136^∗^
s-Cr (mg/dL)	0.74 (0.61-0.88)	0.71 (0.60-0.87)	0.3913^∗^	0.74 (0.61-0.88)	0.71 (0.60-0.87)	0.4962^∗^	0.74 (0.61-0.88)	0.72 (0.60-0.87)	0.6826^∗^
Maximum TG (mg/dL)	271 (215-356)	135 (110-166)	<0.0001^∗^	275 (216-360)	136 (111-167)	<0.0001^∗^	273 (216-357)	136 (110-166)	<0.0001^∗^
Minimum TG (mg/dL)	109 (81-145)	75 (56-102)	<0.0001^∗^	109 (81-145)	76 (57-102)	<0.0001^∗^	109 (81-145)	76 (57-102)	<0.0001^∗^
Mean TG (mg/dL)	183 (145-229)	104 (80-129)	<0.0001^∗^	184 (145-232)	105 (81-129)	<0.0001^∗^	184 (145-230)	105 (81-130)	<0.0001^∗^
SD	60 (47-87)	23 (16-29)	<0.0001^∗^	60 (47-87)	23 (17-30)	<0.0001^∗^	60 (47-87)	23 (16-29)	<0.0001^∗^
Adj-SD	54 (42-78)	21 (15-26)	<0.0001^∗^	55 (42-79)	21 (15-27)	<0.0001^∗^	54 (42-78)	21 (15-26)	<0.0001^∗^
MMD	155 (119-221)	58 (41-74)	<0.0001^∗^	158 (121-221)	58 (42-75)	<0.0001^∗^	157 (120-221)	58 (41-74)	<0.0001^∗^
HDL-C (mg/dL)	51 (43-60)	60 (51-69)	<0.0001^∗^	51 (43-60)	60 (51-69)	<0.0001^∗^	52 (44-61)	59 (49-69)	<0.0001^∗^
LDL-C (mg/dL)	98 (83-119)	97 (84-118)	0.9184^∗^	98 (83-118)	97 (84-118)	0.8720^∗^	98 (83-118)	97 (84-118)	0.7700^∗^
HbA1c (%)	6.6 (6.3-7.3)	6.6 (6.3-7.1)	0.3572^∗^	6.6 (6.3-7.3)	6.6 (6.3-7.1)	0.3671^∗^	6.6 (6.3-7.3)	6.6 (6.3-7.2)	0.6315^∗^
Smoking (current, past, never)	44 (17), 101 (38), 121 (45)	29 (11), 82 (31), 150 (57)	0.0173^∗∗^	43 (17), 99 (38), 118 (45)	30 (11), 84 (31), 153 (57)	0.0186^∗∗^	42 (16), 97 (37), 125 (47)	31 (12), 86 (33), 146 (56)	0.1392^∗∗^
Proteinuria (≥±)	115 (43)	88 (34)	0.0248^∗∗^	113 (43)	90 (34)	0.0214^∗∗^	113 (43)	90 (34)	0.0428^∗∗^
Statins intake	161 (61)	148 (57)	0.3732^∗∗^	159 (61)	150 (56)	0.2464^∗∗^	163 (62)	146 (56)	0.1466^∗∗^
Fibrates intake	11 (4)	1 (0)	0.0039^∗∗^	11 (4)	1 (0)	0.0030^∗∗^	11 (4)	1 (0)	0.0036^∗∗^
Cholesterol transport inhibitors intake	19 (7)	4 (2)	0.0016^∗∗^	19 (7)	4 (2)	0.0011^∗∗^	19 (7)	4 (2)	0.0014^∗∗^

TG: triglyceride; SD: standard deviation; Adj-SD: adjusted SD; MMD: maximum minus minimum difference; BMI: body mass index; sBP: systolic blood pressure; dBP: diastolic blood pressure; eGFR: estimated glomerular filtration rate; s-Cr: serum creatinine; HDL-C: high-density lipoprotein cholesterol; LDL-C: low-density lipoprotein cholesterol; HbA1c: glycated hemoglobin; maximum TG: the highest value of postprandial TG between 2012 and 2015; minimum TG: the lowest value of postprandial TG between 2012 and 2015. ∗Mann–Whitney *U* test. ∗∗Pearson's chi-square test categorical variables are presented as *n* (%), and continuous data are represented as median (interquartile range).

**Table 2:** Multivariate Cox's proportional hazard regression model for the association between postprandial TG variability and eGFR decline (primary endpoint).

HR [95% CI] *p* value	SD ≥ 37	Adj − SD ≥ 34	MMD ≥ 95
Model 1	1.69 [1.15-2.52] 0.0076	1.73 [1.18-2.57] 0.0052	1.64 [1.12-2.43] 0.0110
Model 2	1.61 [1.05-2.51] 0.0276	1.66 [1.09-2.58] 0.0193	1.56 [1.02-2.41] 0.0388
Model 3	1.58 [1.03-2.46] 0.0351	1.63 [1.06-2.52] 0.0251	1.54 [1.01-2.37] 0.0463
Model 4	1.62 [1.05-2.53] 0.0284	1.66 [1.08-2.58] 0.0218	1.59 [1.04-2.47] 0.0340

Model 1: adjusted for age, sex, and BMI. Model 2: adjusted for age, sex, BMI, and mean TG. Model 3: adjusted for age, sex, BMI, mean TG, baseline eGFR, and proteinuria. Model 4: adjusted for age, sex, BMI, mean TG, baseline eGFR, proteinuria, HbA1c, smoking, hypertension, and fibrates intake. TG: triglyceride; SD: standard deviation; Adj-SD: adjusted SD; MMD: maximum minus minimum difference; HR: hazard ratio; 95% CI: 95% confidence intervals; BMI: body mass index; eGFR: estimated glomerular filtration rate; HbA1c: glycated hemoglobin.

**Table 3:** Characteristics of study participants for the secondary analysis with three indices of postprandial TG variability groups (*N* = 181).

Variable	Higher-SD group: SD ≥ 37 (*n* = 91)	Lower-SD group: SD < 37 (*n* = 90)	*p* value	Higher-Adj-SD group: Adj − SD ≥ 34 (*n* = 89)	Lower-Adj-SD group: Adj − SD < 34 (*n* = 92)	*p* value	Higher-MMD group: MMD ≥ 93 (*n* = 91)	Lower-MMD group: SD < 93 (*n* = 90)	*p* value
Age (yr)	67 (60-76)	68 (61-75)	0.3842^∗^	67 (60-75)	68 (61-75)	0.2935^∗^	67 (60-75)	68 (61-76)	0.1994^∗^
Sex (male)	53 (58)	43 (48)	0.1584^∗∗^	52 (58)	44 (48)	0.1531^∗∗^	49 (54)	47 (52)	0.8267^∗∗^
Duration of diabetes (yr)	9 (5-16)	14 (7-19)	0.0287^∗^	10 (5-16)	13 (7-19)	0.0460^∗^	9 (5-15)	14 (7-19)	0.0075^∗^
BMI (kg/m^2^)	23.7 (22.2-25.7)	23.3 (21.3-24.9)	0.0984^∗^	24.0 (22.1-25.8)	23.3 (21.5-24.9)	0.0921^∗^	24.0 (22.1-25.8)	23.3 (21.7-24.8)	0.0674^∗^
sBP (mmHg)	128 (122-136)	132 (124-138)	0.0616^∗^	128 (122-136)	132 (124-138)	0.0894^∗^	128 (122-136)	132 (124-138)	0.0357^∗^
dBP (mmHg)	74 (70-80)	72 (70-78)	0.4281^∗^	74 (70-80)	72 (70-78)	0.4717^∗^	76 (70-80)	72 (70-78)	0.3534^∗^
eGFR (mL/min/1.73 m^2^)	75.0 (63.1-88.1)	75.6 (62.3-87.0)	0.8604^∗^	75.0 (63.1-88.0)	75.6 (62.2-87.1)	0.8403^∗^	75.4 (63.1-88.6)	75.5 (62.3-86.4)	0.7282^∗^
s-Cr (mg/dL)	0.73 (0.60-0.87)	0.69 (0.58-0.81)	0.2107^∗^	0.73 (0.61-0.87)	0.69 (0.58-0.81)	0.2000^∗^	0.71 (0.59-0.87)	0.70 (0.59-0.82)	0.7592^∗^
Maximum TG (mg/dL)	270 (212-343)	135 (113-158)	<0.0001^∗^	274 (212-343)	136 (114-158)	<0.0001^∗^	270 (211-343)	135 (113-158)	<0.0001^∗^
Minimum TG (mg/dL)	109 (86-147)	71 (56-96)	<0.0001^∗^	109 (86-147)	71 (57-96)	<0.0001^∗^	105 (86-147)	72 (56-98)	<0.0001^∗^
Mean TG (mg/dL)	181 (144-233)	99 (80-121)	<0.0001^∗^	182 (145-234)	99 (80-123)	<0.0001^∗^	181 (143-233)	99 (80-126)	<0.0001^∗^
SD	53 (45-80)	24 (17-27)	<0.0001^∗^	54 (46-80)	24 (17-28)	<0.0001^∗^	53 (45-80)	24 (17-27)	<0.0001^∗^
Adj-SD	49 (41-73)	22 (16-25)	<0.0001^∗^	49 (41-73)	22 (16-26)	<0.0001^∗^	49 (41-73)	22 (16-25)	<0.0001^∗^
MMD	144 (115-209)	58 (44-70)	<0.0001^∗^	146 (116-210)	58 (45-70)	<0.0001^∗^	144 (115-209)	58 (44-70)	<0.0001^∗^
HDL-C (mg/dL)	50 (43-60)	60 (49-69)	0.0004^∗^	50 (43-59)	60 (49-69)	0.0004^∗^	51 (45-61)	59 (47-68)	0.0117^∗^
LDL-C (mg/dL)	98 (86-118)	95 (83-115)	0.4922^∗^	98 (86-116)	95 (83-115)	0.5231^∗^	98 (85-115)	96 (83-117)	0.9604^∗^
HbA1c (%)	6.8 (6.4-7.4)	6.6 (6.3-7.0)	0.1717^∗^	6.8 (6.4-7.4)	6.7 (6.3-7.0)	0.1907^∗^	6.8 (6.4-7.4)	6.7 (6.3-7.1)	0.3632^∗^
Smoking (current, past, never)	16 (18), 34 (37), 41 (45)	9 (10), 24 (27), 57 (63)	0.0430^∗∗^	16 (18), 33 (37), 40 (45)	9 (10), 25 (27), 58 (63)	0.0424^∗∗^	15 (16), 33 (36), 43 (47)	10 (11), 25 (28), 55 (61)	0.1680^∗∗^
Albuminuria (mg/dL)	10.6 (6.5-16.5)	8.4 (5.2-14.1)	0.1523^∗^	10.6 (6.3-16.6)	8.5 (5.3-14.0)	0.1727^∗^	10.6 (6.7-16.5)	8.4 (5.1-13.9)	0.0958^∗^
Statins intake	50 (55)	49 (54)	0.9461^∗∗^	49 (55)	50 (54)	0.9238^∗∗^	52 (57)	47 (52)	0.5061^∗∗^
Fibrates intake	5 (5)	0 (0)	0.0241^∗∗^	5 (6)	0 (0)	0.0211^∗∗^	5 (5)	0 (0)	0.0241^∗∗^
Cholesterol transport inhibitors intake	10 (11)	3 (3)	0.0461^∗∗^	10 (11)	3 (3)	0.0378^∗∗^	10 (11)	3 (3)	0.0461^∗∗^

TG: triglyceride; SD: standard deviation; Adj-SD: adjusted SD; MMD: maximum minus minimum difference; BMI: body mass index; sBP: systolic blood pressure; dBP: diastolic blood pressure; eGFR: estimated glomerular filtration rate; s-Cr: serum creatinine; HDL-C: high-density lipoprotein cholesterol; LDL-C: low-density lipoprotein cholesterol; HbA1c: glycated hemoglobin; maximum TG: the highest value of postprandial TG between 2012 and 2015; minimum TG: the lowest value of postprandial TG between 2012 and 2015. ∗Mann–Whitney *U* test. ∗∗Pearson's chi-square test categorical variables are presented as *n* (%), and continuous data are represented as median (interquartile range).

**Table 4:** Multivariate Cox's proportional hazard regression model for the association between postprandial TG variability and the incidence of microalbuminuria.

HR [95% CI] *p* value	SD ≥ 37	Adj − SD ≥ 34	MMD ≥ 93
Model 1	1.60 [1.09-2.36] 0.0170	1.56 [1.06-2.23] 0.0232	1.49 [1.02-2.20] 0.0399
Model 2	1.79 [1.09-2.91] 0.0205	1.73 [1.06-2.83] 0.0295	1.60 [0.98-2.58] 0.0591
Model 3	1.77 [1.08-2.88] 0.0228	1.72 [1.05-2.81] 0.0319	1.58 [0.97-2.56] 0.0657

Model 1: adjusted for age, sex, and BMI. Model 2: adjusted for age, sex, BMI, and mean TG. Model 3: adjusted for age, sex, BMI, mean TG, and baseline eGFR. TG: triglyceride; SD: standard deviation; Adj-SD: adjusted SD; MMD: maximum minus minimum difference; HR: hazard ratio; 95% CI: 95% confidence intervals; BMI: body mass index; eGFR: estimated glomerular filtration rate.

## Data Availability

The data used to support the findings of this study are available from the corresponding author upon request.

## Abbreviations

eGFR: Estimated glomerular filtration rate
DM: Diabetes mellitus
TG: Triglyceride
SD: Standard deviation
Adj-SD: Adjusted SD
MMD: Maximum and minus minimum difference
HR: Hazard ratio
CI: Confidence intervals
ESRD: End-stage renal disease
DKD: Diabetic kidney disease
GFR: Glomerular filtration rate
HDL-C: High-density lipoprotein cholesterol
UACR: Urine albumin-to-creatinine ratio
BMI: Body mass index
HbA1c: Glycated hemoglobin
LDL-C: Low-density lipoprotein cholesterol
CKD: Chronic kidney disease.
